# First-in-Human Evaluation of the Safety, Tolerability, and Pharmacokinetics of SPR720, a Novel Oral Bacterial DNA Gyrase (GyrB) Inhibitor for Mycobacterial Infections

**DOI:** 10.1128/AAC.01208-21

**Published:** 2021-10-18

**Authors:** Angela K. Talley, Archie Thurston, Grayson Moore, Vipul K. Gupta, Myriah Satterfield, Erika Manyak, Suzanne Stokes, Aaron Dane, David Melnick

**Affiliations:** a Spero Therapeutics, Inc., Cambridge, Massachusetts, USA; b ADME Solutions, Inc., San Diego, California, USA; c DaneStat Consulting, Macclesfield, United Kingdom

**Keywords:** DNA gyrase inhibitor, aminobenzimidazole, nontuberculous mycobacteria, *Mycobacterium avium* complex, *Mycobacterium tuberculosis*, pharmacokinetics, SPR720, DNA gyrase inhibitor

## Abstract

SPR720 (phosphate prodrug of SPR719) is a novel aminobenzimidazole bacterial DNA gyrase (GyrB) inhibitor in development for nontuberculous mycobacterial pulmonary disease (NTM-PD) and pulmonary tuberculosis. SPR719 has demonstrated activity against clinically relevant mycobacteria *in vitro* and in murine and hollow-fiber infection models. This phase 1 randomized, double-blind, placebo-controlled, single ascending dose (SAD)/multiple ascending dose (MAD) trial evaluated the safety, tolerability, and pharmacokinetics of SPR720/SPR719. A total of 96 healthy volunteers (*n* = 8/cohort, 3:1 randomization) received SPR720 (or placebo) as single oral doses ranging from 100 to 2,000 mg or repeat total daily doses ranging from 500 to 1,500 mg for 7 or 14 days. SPR720 was well tolerated at daily doses of up to 1,000 mg for up to 14 days. Across SAD/MAD cohorts, the most common adverse events (AEs) were gastrointestinal (nausea, vomiting, and diarrhea) and headache, all of mild or moderate severity and dose dependent. No serious AEs were reported. The median SPR719 *T*_max_ ranged from 2.8 to 8.0 h across cohorts, and the *t*_1/2_ ranged from 2.9 to 4.5 h and was shown to be dose independent. Dosing with food decreased SPR719 plasma exposure by approximately 20%. In the MAD cohorts, SPR719 plasma exposure declined approximately 40% between days 1 and 7, suggesting induction of an elimination pathway. However, plasma AUC_0–24_ was comparable between days 7 and 14. The results of this first-in-human study suggest that predicted therapeutic exposures of SPR719 can be attained with a once-daily oral administration of SPR720. (This study has been registered at ClinicalTrials.gov under registration no. NCT03796910.)

## TEXT

Nontuberculous mycobacterial (NTM) pulmonary infection causes a chronic, progressive lung disease (NTM-PD) that occurs through inhalation of mycobacteria from environmental sources, especially in water and soil (1). Clinically relevant NTM species include Mycobacterium avium complex (MAC; consisting of M. avium, *M. intracellulare*, and *M. chimaera*), M. abscessus, M. xenopi, and M. kansasii (1). The pathophysiology of NTM-PD is poorly understood. In 2018, an estimated 75,000 to 105,000 people in the United States were diagnosed with NTM lung infection, with a higher frequency in older adults, and particularly in older nonsmoking women and men with COPD (2, 3). The prevalence of NTM-PD is increasing worldwide and is associated with increased health care costs and poor outcomes (1, 4–11).

No oral antimicrobial agents are currently approved for the treatment of NTM pulmonary infections, so current treatment guidelines are based on available clinical trial data for existing antibiotics (12). Recommended regimens vary by pathogen species and are guided by *in vitro* susceptibility testing. Current treatment guidelines for NTM-PD recommend prolonged treatment for at least 12 months after culture conversion with a three-drug approach comprised of azithromycin and two other drugs (rifampin and ethambutol, streptomycin, or intravenous amikacin) (12, 13). Even in the setting of prolonged combination therapy, outcomes are poor and often complicated by treatment-limiting adverse effects, resulting in high relapse and mortality rates (4, 14–17). New effective treatment options for NTM are needed.

SPR720 is a chemically stable phosphate prodrug that converts rapidly to SPR719, the active moiety, *in vivo*. It belongs to a novel class of antibacterial agents, the aminobenzimidazoles, which targets the ATPase subunits of bacterial DNA gyrase and, when present, topoisomerase, by a mechanism which is distinct from that of the fluoroquinolones. SPR720 is in clinical development for the treatment of NTM-PD and pulmonary tuberculosis (18). SPR719 has potent antimycobacterial activity comparable to clarithromycin and rifampin across clinically relevant mycobacterial species *in vitro*, including M. avium (19), M. kansasii, M. ulcerans, M. marinum, and *M. chimaera* (20, 21), with no evidence of cross-resistance to other standard-of-care agents (20–22). The efficacy of SPR719 against these mycobacterial species has been further established in murine models of infection and hollow-fiber studies with demonstrated additive activity of SPR719 in combination with clarithromycin and ethambutol (22–24). Preclinical studies to date support the further clinical evaluation of SPR720 as a candidate oral antimicrobial agent for the treatment of NTM-PD and pulmonary tuberculosis. We report here the results from a first-in-human study of SPR720 evaluating the safety, tolerability, and pharmacokinetics (PK) of SPR720/SPR719 using a single ascending dose (SAD)/multiple ascending dose (MAD) in healthy volunteers.

## RESULTS

A total of 96 subjects were randomized, 56 in the SAD phase and 40 in the MAD phase. Baseline characteristics were comparable between treatment groups for both SAD and MAD phases (Table 1), except for a higher mean age in the elderly cohort of the SAD phase. The majority of subjects were male and Caucasian. All subjects completed the study and were included in the safety and PK analyses.

**TABLE 1:** Baseline characteristics for SAD and MAD phases

Parameter	SAD phase		MAD phase	
	Placebo (*n* = 14)	SPR720 (*n* = 42)	Placebo (*n* = 10)	SPR720 (*n* = 30)
Mean age (yr) ± SD^*a*^	40.1 ± 14.6	39.0 ± 16.0^*a*^	32.9 ± 12.1	35.4 ± 9.5
No. male (%)	12 (85.7)	36 (85.7)	9 (90.0)	27 (90.0)
Mean body wt (kg) ± SD	81.8 ± 9.4	80.6 ± 10.5	79.0 ± 9.7	78.4 ± 9.6
Mean BMI^*b*^ (kg/m^2^) ± SD	26.5 ± 2.7	26.5 ± 2.8	25.8 ± 3.4	25.2 ± 3.0
No. (%) Caucasian	14 (100.0)	42 (100.0)	8 (80.0)	30 (100.0)

a The mean age ± the SD for the elderly cohort was 67.8 ± 2.6 years.

b BMI, body mass index.

### Safety/tolerability.

Across SAD cohorts, 21 (37.5%) subjects reported 40 treatment-emergent adverse events (TEAEs). Of the 42 SAD subjects who received SPR720, 18 (42.9%) reported 35 TEAEs. A higher proportion of subjects reported TEAEs in the 1,500-mg and 2,000-mg dose groups (66.7 and 100.0%, respectively) compared to the lower-dose 100- to 1,000-mg cohorts (16.7 to 33.3%) (Table 2). All TEAEs were of mild or moderate severity. The most common TEAEs were gastrointestinal (nausea, vomiting, and diarrhea) and headache. No serious TEAEs were reported, and no deaths occurred.

**TABLE 2:** Incidence of treatment-emergent adverse events in SAD and MAD groups

Preferred term	No. (%) of subjects									
	Placebo, overall (*n* = 14)	Cohort 1, 100 mg (*n* = 6)	Cohort 2, 250 mg (*n* = 6)	Cohort 3, 500 mg (*n* = 6)	Cohort 4, 1,000 mg, fasted (*n* = 6)	Cohort 4, 1,000 mg, fed (*n* = 6)	Cohort 5, 1,500 mg (*n* = 6)	Cohort 6, 2,000 mg (*n* = 6)	Elderly, 500 mg (*n* = 6)	Overall, SPR720 (*n* = 42)
SAD										
Any TEAE	3 (21.4)	1 (16.7)	2 (33.3)	1 (16.7)	1 (16.7)	0	4 (66.7)	6 (100)	3 (50.0)	18 (42.9)
Nausea	0	0	0	0	0	0	4 (66.7)	5 (83.3)	0	9 (21.4)
Headache	1 (7.1)	0	0	0	1 (16.7)	0	3 (50.0)	3 (50.0)	0	7 (16.7)
Vomiting	0	0	0	0	0	0	3 (50.0)	5 (83.3)	0	8 (19.0)
Diarrhea	0	0	0	1 (16.7)	0	0	0	2 (33.3)	0	3 (7.1)
Abdominal discomfort	0	0	1 (16.7)	0	0	0	0	0	0	1 (2.4)
Amylase increased	0	0	0	0	0	0	0	0	1 (16.7)	1 (2.4)
Dyspepsia	0	0	0	0	0	0	0	0	1 (16.7)	1 (2.4)
Neuralgia	0	0	0	0	0	0	0	0	1 (16.7)	1 (2.4)
Rhinorrhea	0	0	0	0	0	0	0	0	1 (16.7)	1 (2.4)
Skin abrasion	0	1 (16.7)	0	0	0	0	0	0	0	1 (2.4)
Toothache	0	0	1 (16.7)	0	0	0	0	0	0	1 (2.4)
										

	Placebo, overall (*n* = 10)	Cohort 1, 500 mg, 7 days (*n* = 6)	Cohort 2, 1,000 mg, 7 days (*n* = 6)	Cohort 3, 1,500 mg (750 mg q12h), 7 days (*n* = 6)	Cohort 4, 500 mg, 14 days (*n* = 6)	Cohort 5, 1,000 mg, 14 days (*n* = 6)	Overall, MAD, SPR720 (*n* = 30)			
MAD										
Any TEAE	1 (10.0)	4 (66.7)	6 (100)	4 (66.7)	1 (16.7)	3 (50.0)	18 (60.0)			
Diarrhea	1 (10.0)	2 (33.3)	4 (66.7)	4 (66.7)	0	3 (50.0)	13 (43.3)			
Headache	0	2 (33.3)	3 (50.0)	2 (33.3)	1 (16.7)	0	8 (26.7)			
Nausea	0	2 (33.3)	1 (16.7)	3 (50.0)	0	1 (16.7)	7 (23.3)			
Vomiting	0	1 (16.7)	2 (33.3)	1 (16.7)	0	0	4 (13.3)			
Abdominal pain	0	0	2 (33.3)	1 (16.7)	0	0	3 (10.0)			
Back pain	0	0	1 (16.7)	0	0	1 (16.7)	2 (6.7)			
Abdominal discomfort	0	0	1 (16.7)	0	0	0	1 (3.3)			
Abdominal pain upper	0	0	0	0	0	1 (16.7)	1 (3.3)			
Contusion	0	0	1 (16.7)	0	0		1 (3.3)			
Gastroesophageal reflux disease	0	0	0	0	0	1 (16.7)	1 (3.3)			
Insomnia	0	1 (16.7)	0	0	0	0	1 (3.3)			
Lipase increased	0	0	0	0	0	1 (16.7)	1 (3.3)			
Oropharyngeal pain	0	0	1 (16.7)	0	0	0	1 (3.3)			
Pancreatic enzymes increased	0	0	0	1 (16.7)	0	0	1 (3.3)			
Rash	0	0	0	1 (16.7)	0	0	1 (3.3)			
Syncope	0	1 (16.7)	0	0	0	0	1 (3.3)			

Across MAD cohorts, 19 (47.5%) subjects reported 113 TEAEs. Of the 30 MAD subjects who received SPR720, 18 (60.0%) reported a total of 101 TEAEs (Table 2). A higher proportion of subjects reported TEAEs in the 7-day dose cohorts (66.7, 100, and 66.7% for the 500-, 1,000-, and 1,500-mg groups, respectively) than in the 14-day cohorts (16.7 and 50% for the 500- and 1,000-mg groups, respectively), which likely represents improved tolerability with continued dosing. All TEAEs were of mild to moderate severity and resolved without treatment. The most common TEAEs were gastrointestinal (diarrhea, nausea, and vomiting) and headache. No serious TEAEs were reported, and no deaths occurred.

One subject in the MAD 1,500-mg (750 mg q12h) dose group terminated dosing on day 6 due to a transient elevation in pancreatic enzymes reported as a moderate (grade 2 [CTCAE]) AE but remained in the study for follow-up visits. Peak values of amylase were 226 U/liter (2.3× upper limit of normal [ULN]) and lipase were 427 U/liter (7.1× ULN) on study day 5. Trough plasma SPR719 concentrations exceeded 1,000 ng/ml in this subject throughout the dosing period (range, 4,580 to 1,680 ng/ml from day 2 through day 5). The subject was asymptomatic, and clinical laboratory findings resolved without treatment. Transient elevations in alanine aminotransferase (ALT) levels (>1.5× ULN to <3× ULN) were observed in three subjects from the 14-day cohorts (500 and 1,000 mg); but no cases of Hy’s Law occurred. There were no clinically significant changes in vital signs or 12-lead electrocardiogram (ECG) parameters during the study.

### Pharmacokinetics.

Due to rapid and extensive conversion of SPR720 to active moiety SPR719 *in vivo*, plasma concentrations of SPR720 across all cohorts were low and irregular following oral administration of SPR720. Therefore, characterization of SPR720 PK were best represented by assessment of SPR719 PK parameters.

**FIG 1:**
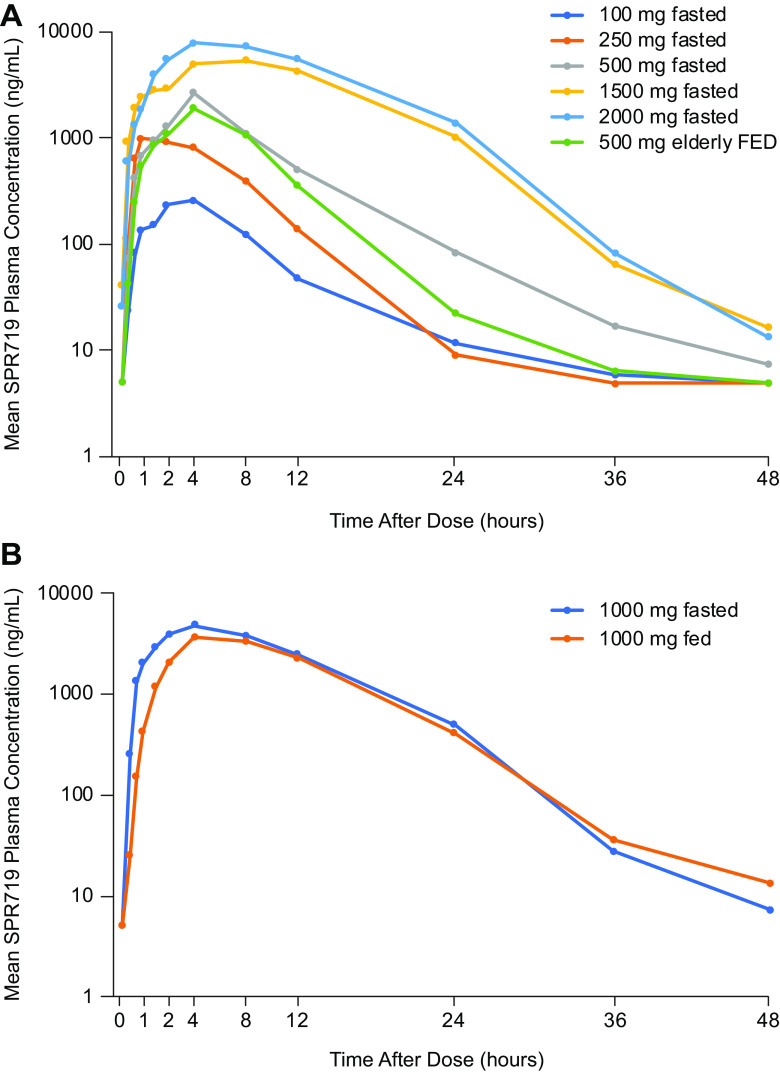
Geometric mean plasma SPR719 concentration-time curves following single ascending doses (SAD) of SPR720 (A) and SP720 of 1,000 mg during fed and fasting states (B).

**(i) Single ascending dose.** In the SAD phase, mean plasma SPR719 concentrations increased with increasing dose of SPR720 (Fig. 1). The PK parameters of SPR719 are shown in (Table 3). The median *T*_max_ for SPR719 ranged from 2.8 to 8.0 h across cohorts, and the mean elimination half-life (*t*_1/2_) ranged from 2.9 h to 4.5 h. A dose-proportional increase in SPR719 *C*_max_ and a greater-than-dose-proportional increase in SPR719 AUC were observed with increasing doses of SPR720 (Table 4). Administration of SPR720 following a high-fat meal resulted in a decrease of approximately 20% in geometric mean SPR719 plasma *C*_max_, AUC_last_, and AUC_inf_ compared to the fasting state after a single 1,000-mg dose (Table 5). The effect of age on SPR719 PK following a single 500-mg dose of SPR720 was examined in healthy elderly subjects ≥65 years (SAD cohort 7) versus subjects <65 years in the SAD cohort 2. In the elderly versus younger subjects, geometric mean plasma SPR719 *C*_max_ increased approximately 1.8-fold (1,760 ng/ml versus 2,860 ng/ml, respectively), and geometric mean AUC_0–24_ increased approximately 1.5-fold (18,300 ng ⋅ h/ml versus 21,000 ng ⋅ h/ml, respectively). These data suggest a trend toward higher exposure of SPR719 in healthy elderly subjects. Urinary excretion of unchanged SPR719 was minimal (<0.5%) across SAD cohorts.

**TABLE 3:** Summary of plasma PK parameters of SPR719 following single oral dose administration of SPR720 in healthy volunteers

SPR720 treatment (*n*)	Mean (% CV)^*a*^						
	*C*_max_ (ng/ml)	*T*_max_ (h)	*t*_1/2_ (h)	AUC_last_ (h ⋅ ng/ml)	AUC_0–24_ (h ⋅ ng/ml)	AUC_inf_ (h ⋅ ng/ml)	AUC_ext_ (%)
100 mg (6)	278 (58.3)	3 (1–4)	3.15 (65.4)^*b*^	1,360 (95.6)	1,620 (174.6)^*b*^	1,650 (184.0)^*b*^	3.82 (132.0)^*b*^
250 mg (6)	1,220 (74.1)	2.75 (1–4)	2.92 (23.4)^*c*^	6,060 (44.7)	6,900 (41.3)^*c*^	6,940 (40.8)^*c*^	2.68 (93.7)^*c*^
500 mg (6)	2,860 (28.4)	4 (2–4)	3.59 (42.7)^*d*^	16,000 (58.3)	21,000 (35.6)^*d*^	21,600 (39.9)^*d*^	1.04 (88.7)^*d*^
1,000 mg (6)	4,730 (22.1)	4 (1.5–4)	3.96 (15.2)^*d*^	56,500 (22.6)	54,700 (21.6)	56,800 (22.3)^*d*^	0.234 (211.8)^*d*^
1,000 mg (6)	4,000 (16.4)	6 (4–8)	4.31 (25.2)	45,100 (27.4)	43,300 (25.7)	45,200 (27.3)	0.242 (76.3)
1,500 mg (6)	5,990 (39.8)	8 (1–12)	4.50 (24.2)^*d*^	75,700 (49.5)	72,300 (47.3)	81,600 (37.8)^*d*^	0.161 (107.7)^*d*^
2,000 mg (6)	8,240 (33.6)	4 (2–8)	3.96 (18.0)	111,000 (27.6)	106,000 (27.4)	112,000 (27.5)	0.215 (87.7)
500 mg, elderly (6)	1,760 (73.8)	4 (2–8)	3.36 (40.6)^*e*^	11,400 (75.0)	18,300 (55.0)^*b*^	24,900 (8.8)^*e*^	0.307 (36.6)^*e*^

a AUC_0–24_, area under the concentration-time curve from time zero to 24 h; AUC_ext_, area under the concentration-time curve extrapolated to infinity; AUC_inf_, area under the concentration-time curve from time zero to infinity; AUC_last_, area under the concentration-time curve to the last measured time point; *C*_max_, maximum concentration; *t*_1/2_, elimination half-life; *T*_max_, time of maximum observed concentration. *C*_max_ and AUC values are expressed as geometric means (% coefficient of variation [CV%]), *T*_max_ values are expressed as medians (ranges), and half-life (*t*_1/2_) values are expressed as arithmetic means. The lower limit of quantitation is 10 ng/ml; BQL assigned a value of missing.

b *n* = 3.

c *n* = 5.

d *n* = 4.

e *n* = 2.

**TABLE 4:** Statistical analysis of SPR719 dose proportionality in the SAD phase (fasted)^*a*^

Parameter	Geometric LS mean						Slope (95% CI)
	100 mg (*n* = 6)	250 mg (*n* = 6)	500 mg (*n* = 6)	1,000 mg, fasted (*n* = 6)	1,500 mg (*n* = 6)	2,000 mg (*n* = 6)	
*C*_max_ (ng/ml)	278	1,220	2,860	4,730	5,990	8,240	1.08 (0.93–1.23)
AUC_last_ (h ⋅ ng/ml)	1,360	6,060	16,000	56,500	75,700	111,000	1.48 (1.33–1.64)
AUC_inf_ (h ⋅ ng/ml)	1,650	6,940	21,600	56,800	81,600	112,000	1.40 (1.22–1.58)

a Results for *C*_max_ and AUC were obtained using a regression analysis on log-transformed values versus log-transformed dose using the power model (including an additional term for fed/fasted status for the model incorporating all cohorts for part 2). AUC_inf_, area under the concentration-time curve (AUC) extrapolated to infinity; AUC_last_, AUC from time of dosing to time of the last measurable concentration; CI, confidence interval; *C*_max_, maximum plasma concentration.

**TABLE 5:** Statistical analysis of food effect on the PK of SPR719 following single oral 1,000 mg doses of SPR720^*a*^

Parameter	Geometric LS mean		Geometric LS mean ratio (%) 90% CI	Within-subject CV (%)
	1,000 mg, fasted (*n* = 6)	1,000 mg, fed (*n* = 6)		
*C*_max_ (ng/ml)	4,730	4,000	84.6 (69.9–102.4)	16.6
AUC_last_ (h ⋅ ng/ml)	56,500	45,100	79.9 (68.5–93.0)	13.2
AUC_inf_ (h ⋅ ng/ml)	56,800	45,200	79.6 (68.3–92.8)	13.2

a Results were obtained using a fixed-effects ANOVA with fixed effects of treatment and subject. AUC_inf_, area under the concentration-time curve (AUC) extrapolated to infinity; AUC_last_, AUC from time of dosing to time of the last measurable concentration; CI, confidence interval; *C*_max_, maximum plasma concentration; CV, coefficient of variation; LS mean, least-squares mean.

**FIG 2:**
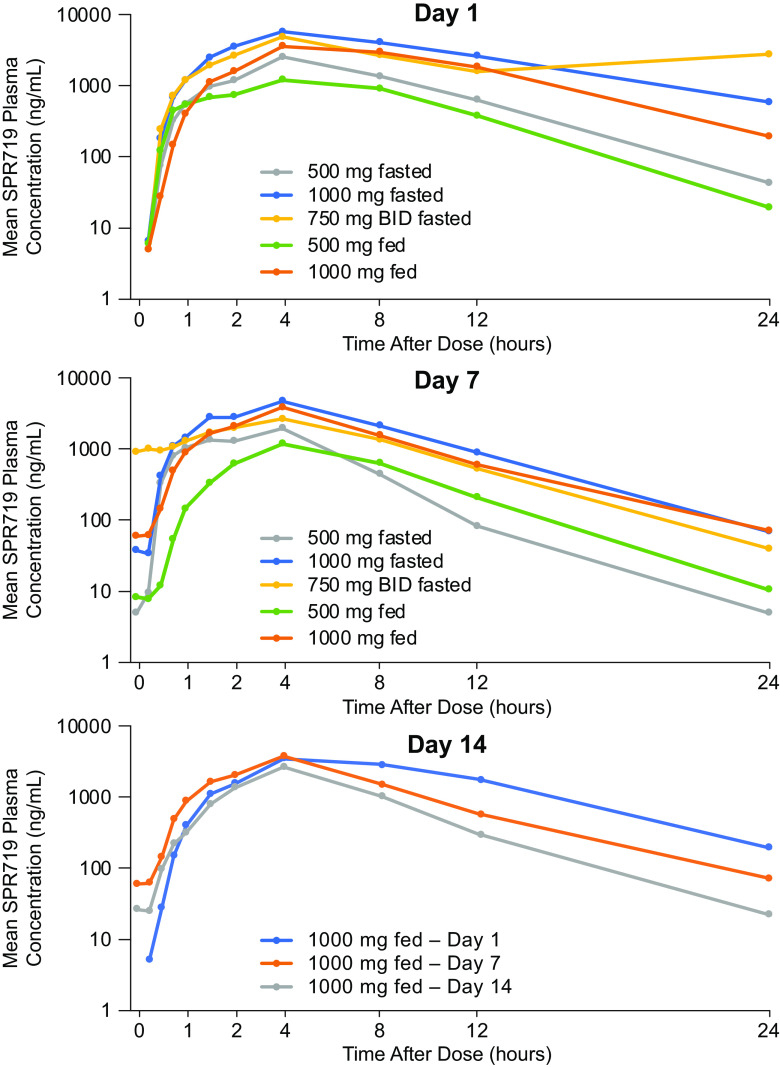
Geometric mean plasma SPR719 concentration-time curves following multiple ascending doses (MAD) of SPR720 on days 1, 7, and 14.

**(ii) Multiple ascending dose.** In the MAD phase, SPR719 plasma concentrations increased with increasing dose of SPR720 (Fig. 2). The PK profile of SPR719 was characterized by a median *T*_max_ of 4 h and a mean *t*_1/2_ that ranged from 1.7 to 5.1 h (Table 6). Dose-proportionality analysis showed that the SPR719 plasma *C*_max_ and AUC_0–24_ increased in a greater-than-dose-proportional manner with increasing doses of SPR720 (Table 7). Repeat administration of SPR720 was associated with a decrease in plasma exposure of SPR719 over 14 days. For the 500- and 1,000-mg 7-day dose groups, the geometric mean SPR719 plasma *C*_max_ was similar at day 1 compared to day 7, with a <10% decrease from day 1 to day 7. However, for the 1,500-mg (750 mg q12h) 7-day group, geometric mean *C*_max_ decreased approximately 44% from day 1 to day 7. The geometric mean plasma SPR719 plasma AUC decreased by 41 to 46% from day 1 to day 7 across these three cohorts. Similarly, in the 14-day 500- and 1,000-mg (nonfasting) dose groups (MAD cohorts 4 and 5), the geometric mean SPR719 plasma AUC decreased by 34 to 40% from day 1 to day 7 (Table 8 and Fig. 2). However, between day 7 and day 14, the geometric mean SPR719 plasma AUC decreased by 10% for the 500-mg group and by 33% for the 1,000-mg group, suggesting that any induction of SPR719 elimination pathways was stabilizing between days 7 and 14. Trough concentrations of SPR719 remained stable and low between days 7 and 14 for the 500- and 1,000-mg groups. In contrast, trough concentrations of SPR719 with more frequent dosing (750 mg q12h) were notably higher (7.7- and 21.7-fold on days 2 and 7, respectively) versus that observed with 1,000-mg once-daily dosing (Table 9). Similar to the SAD cohort, excretion of unchanged SPR719 in urine (0 to 24 h) was low (<1.5%) after multiple doses of SPR720 for up to 14 days.

**TABLE 6:** Summary of SPR719 PK parameters following oral administration of SPR720 in healthy volunteers: MAD phase

SPR720 treatment (*n*)	Day	Mean (% CV)^*a*^						
		*C*_max_ (ng/ml)	*T*_max_ (h)	*t*_1/2_ (h)	AUC_0–12_ (h ⋅ ng/ml)	AUC_0–24_ (h ⋅ ng/ml)	AUC_inf_ (h ⋅ ng/ml)	Accumulation ratio
500 mg QD, 7-day dose group (6)	1	2,470 (40.8)	4.0 (4–8)	NC	14,700 (41.5)	16,800 (47.2)	NC	NA
	7	2,420 (39.9)	4 (1.5–4)	1.67 (11.8)	9,480 (25.8)	9,970 (24.7)	10,900 (19.7)	0.593 (52.8)
1,000 mg QD, 7-day dose group (6)	1	5,610 (20.1)	4 (4–4)	NC	43,200 (24.5)	57,000 (34.9)	NC	NA
	7	5,110 (21.4)	4 (1.5–4)	2.87 (14.3)	27,400 (34.3)	30,600 (40.7)	33,300 (40.2)	0.537 (44.4)
1,500 mg total daily dose (750 mg q12h), 7-day dose group (6)	1	4,690 (24.0)	4 (4–4)	NC	31,300 (26.0)	54,700 (39.7)	NC	NA
	7	2,520 (34.8)	4 (4–4)	4.28 (70.8)	17,300 (41.2)	19,300 (44.0)^*b*^	19,700 (42.1)	0.386 (17.8)
500 mg QD, 14-day dose group (6)	1	1,410 (48.5)	4 (1–8)	NC	8,140 (68.4)	9,420 (75.7)	NC	NA
	7	1,140 (72.8)	4 (1.5–8)	NC	5,680 (97.0)	6,260 (100.6)	NC	NC
	14	967 (78.6)	4 (1–8)	5.05 (81.0)	5,040 (93.8)	5,640 (94.1)	9,720 (50.8)	0.599 (103.9)
1,000 mg QD, 14-day dose group (6)	1	3,500 (28.6)	4 (4–8)	NC	27,500 (30.6)	35,700 (25.8)	NC	NA
	7	3,490 (56.0)	4 (4-4)	NC	19,400 (63.6)	21,600 (68.7)	NC	NC
	14	2,500 (51.6)	4 (4–8)	3.67 (53.0)	13,300 (42.5)	14,500 (43.1)	19,900 (24.9)	0.405 (67.3)

a *C*_max_ and AUC are expressed as geometric means, *T*_max_ values are expressed as medians (ranges), and half-life values are expressed as arithmetic means. AUC_0–12_, area under the concentration-time curve from time zero to 12 h; AUC_0–24_, area under the concentration-time curve from time zero to 24 h; AUC_inf_, area under the concentration-time curve from time zero to infinity; *C*_max_, maximum concentration; *t*_1/2_, elimination half-life; *T*_max_, time of maximum observed concentration; NC, not calculable. Dosing of SPR720 in the 7-day dose groups was administered in the fasting state; dosing in the 14-day dose groups was administered without regard to meals.

b The AUC_0–24_ cannot be compared between day 1 and day 7 for cohort 3 (750 mg BID) since the subjects received two doses (750 mg q12 h = 1,500 mg) on day 1 but only one dose (750 mg once in the morning) on day 7.

**TABLE 7:** Statistical analysis of SPR719 dose proportionality in the MAD phase^*a*^

Treatment day and group	Parameter	Geometric LS mean			Slope (95% CI)
		SPR720, 500 mg (*n* = 6)	SPR720, 1,000 mg (*n* = 6)	SPR720, 750 mg q12h (*n* = 6)	
Day 1, fasted	*C*_max_ (ng/ml)	2,470	5,610	NA	1.18 (0.60–1.76)
Day 7, fasted	*C*_max_ (ng/ml)	2,420	5,110	NA	1.08 (0.50–1.65)
	AUC_0–12_ (h ⋅ ng/ml)	9,480	27,400	NA	1.53 (0.98–2.08)
Day 1, fed	*C*_max_ (ng/ml)	1,410	3,500	4,690	1.42 (0.60–2.25)
Day 7, fed	*C*_max_ (ng/ml)	1,140	3,490	2,520	1.64 (0.72–2.55)
	AUC_0–12_ (h ⋅ ng/ml)	5,680	19,400	17,300	1.83 (0.70–2.97)
Day 14, fed	*C*_max_ (ng/ml)	967	2,500	NA	1.37 (0.26–2.49)
	AUC_0–12_ (h ⋅ ng/ml)	5,040	13,300	NA	1.40 (0.23–2.57)

a Results for *C*_max_ and AUCs were obtained using a regression analysis on log-transformed values versus log-transformed dose using the power model (including an additional term for fed/fasted status for the model incorporating all cohorts for part 2). AUC_0–12_, area under the concentration-time curve (AUC) from time zero to 12 h postdose; C, cohort; CI, confidence interval; *C*_max_, maximum plasma concentration; LS mean, least-squares mean; NA, not applicable. Dosing of SPR720 in the 7-day dose groups was administered in the fasting state; dosing in the 14-day dose groups was administered without regard to meals.

**TABLE 8:** Summary of statistical analysis of SPR719 accumulation in MAD phase^*a*^

SPR720 group	Parameter	Geometric LS mean			Geometric LS mean accumulation ratio (%) (90% CI)	
		Day 1	Day 7	Day 14	Day 7/day 1	Day 14/day 1
500 mg (*n* = 6)	*C*_max_ (ng/ml)	2,470	2,420		97.7 (73.9–129.3)	NA
	AUC_0–24_ (h ⋅ ng/ml)	16,800	9,970		59.3 (39.4–89.2)	NA
1,000 mg (*n* = 6)	*C*_max_ (ng/ml)	5,610	5,100		90.9 (74.4–111.1)	NA
	AUC_0–24_ (h ⋅ ng/ml)	57,000	30,600		53.7 (37.8–76.1)	NA
1,500 mg (750 mg q12h) (*n* = 6)	*C*_max_ (ng/ml)	4,490	2,520		56.1 (47.3–66.5)	NA
	AUC_0–12_ (h ⋅ ng/ml)	29,200	17,300		59.2 (46.2–75.9)	NA
500 mg (*n* = 6)	*C*_max_ (ng/ml)	1,410	1,140	967	80.9 (48.5–135.2)	68.7 (38.3–123.3)
	AUC_0–24_ (h ⋅ ng/ml)	9,420	6,260	5,640	66.4(31.8–138.7)	59.9 (29.6–121.0)
1,000 mg (*n* = 6)	*C*_max_ (ng/ml)	3,500	3,490	2,500	99.9 (61.4–162.7)	71.6 (45.1–113.6)
	AUC_0–24_ (h ⋅ ng/ml)	35,700	21,600	14,500	60.4 (34.8–105.0)	40.5 (27.2–60.4)

a Mixed-effects ANOVA was performed on log-transformed data with the fixed effect of study day and a random effect of subjects. Parameter estimates for AUC_0–24_ were calculated as a partial area based on the actual predose sampling time of the following day, where a bleed time deviation occurred at the 24-h time point and the *k*_el_ was not calculable. ANOVA, analysis of variance; AUC_0–12_, area under the concentration-time curve (AUC) from time zero to 12 h postdose; AUC_0–24_, AUC from time zero to 24 h postdose; CI, confidence interval; *C*_max_, maximum plasma concentration; LS mean, least-squares means; NA, not applicable. Dosing of SPR720 in the 7-day dose groups was administered in the fasting state; dosing in the 14-day dose groups was administered without regard to meals.

**TABLE 9:** Trough concentrations of SPR719 by dose in the MAD phase

Dose group	Geometric LS mean trough concn (ng/ml) at day^*a*^:												
	2	3	4	5	6	7	8	9	10	11	12	13	14
500 mg × 7 days	20.8	9.7	7.1	5.7	5.0	5.0	NA	NA	NA	NA	NA	NA	NA
1,000 mg × 7 days	306	100	34.7	18.1	21.1	18.7	NA	NA	NA	NA	NA	NA	NA
750 mg q12h × 7 days	2,370	1,510	1,430	1,300	662	406	NA	NA	NA	NA	NA	NA	NA
500 mg × 14 days	12.4	8.5	7.2	7.0	6.6	7.2	8.6	5.7	8.1	11.5	8.3	5.0	7.4
1,000 mg × 14 days	176	85	42.7	50.2	54.4	25.5	25.3	22.1	18.7	20.4	14.2	10.0	12.0

a NA, not applicable.

## DISCUSSION

A total of 96 subjects (including 8 healthy elderly subjects, age ≥65 years) were randomized and received study drug. SPR720 was well tolerated at daily doses up to 1,000 mg over the maximum duration of 14 days. Across SAD/MAD cohorts, the most common adverse events (AEs) were gastrointestinal (nausea, vomiting, and diarrhea) and headache, all of mild or moderate severity and dose dependent. No serious AEs were reported. While slight elevations in ALT occurred in 3 subjects with 14 days of dosing, these were reversible and not clinically significant. There was a single discontinuation adverse event related to an asymptomatic elevation in amylase and lipase in a subject receiving 750 mg of SPR720 twice daily, although there was no associated clinical or ultrasound evidence of acute pancreatitis.

Across SAD cohorts, minimal to low levels of the prodrug (SPR720) were observed, indicating efficient conversion of SPR720 to the active moiety, SPR719. *In vitro* studies using intestinal and liver S9 fractions have indicated that SPR720 is rapidly hydrolyzed to the active moiety SPR719. Intestinal alkaline phosphatase is reported to cleave phosphate ester prodrugs and is probably responsible for the conversion of SPR720 to SPR79. There were dose-proportional and greater-than-dose-proportional increases in the SPR719 plasma *C*_max_ and AUC_0–24_, respectively, following oral administration of SPR720 at 100 to 2,000 mg. Food decreased SPR719 exposure by approximately 20%, but this was not considered clinically meaningful.

In MAD cohorts, SPR719 plasma AUC declined approximately 40% between day 1 and day 7. This is indicative of potential induction of an elimination pathway. However, SPR719 plasma AUC_0–24_ was similar at days 7 and 14, suggesting that induction had stabilized after day 7 after multiple dose administration of SPR720. This conclusion is further supported by SPR719 trough plasma concentrations, which also stabilized in the 14-day MAD cohorts (cohorts 4 and 5) by the end of dosing (Table 9).

Urinary excretion of SPR719 was low (<1.5% after both single and multiple oral doses), indicating that renal elimination of unchanged SPR179 is a minor pathway of disposition. Metabolic clearance appears to be the primary route of elimination for SPR720/719. Presystemic conversion of the phosphate prodrug SPR720 to the active moiety SPR719 is supported by data from the present study, in which plasma concentrations of SPR720 across all SAD and MAD cohorts were low across subjects and cohorts. Based on preclinical studies and studies in hepatic microsomes, the proposed metabolic pathway of SPR719 in humans is dealkylation, hydroxylation, or glucuronidation. An *in vitro* CYP-phenotyping study with recombinant human enzymes indicated that SPR719 is predominantly metabolized by CYP3A4. Since the standard of care for NTM-PD is combination antibiotic therapy, the potential for drug-drug interactions with other anti-NTM agents such as the macrolides and rifamycins will need to be assessed.

One of the MAD cohorts also evaluated a 750-mg q12h dosing regimen for SPR720. Increasing the dosing frequency from once daily to q12h resulted in a substantial increase in the trough concentrations of SPR719 and SPR719, supporting once-daily dosing of SPR720. However, further PK and PD evaluations are warranted.

Nonclinical studies demonstrated that SPR719 is efficacious in an M. avium hollow-fiber infection model (HFIM) using a PK profile that supports once-daily dosing in humans (23). SPR720 also demonstrated efficacy in a chronic murine model of M. avium pulmonary infection, both alone and in combination with standard-of-care agents, resulting in statistically significant reduction in bacterial burden following the oral administration of SPR720 once daily (24). These data support the further development of SPR720 for the treatment of NTM-PD in humans.

Recommendations for the treatment of infections caused by NTM are based mostly on anecdotal data or expert opinion, with limited good-quality data available from randomized, controlled studies (5, 13, 25). Standard-of-care oral treatment includes azithromycin, rifampin, ethambutol, isoniazid, fluoroquinolones, doxycycline, linezolid, and trimethoprim-sulfamethoxazole (5, 12, 13). The administration of combinations of these drugs is required for treatment durations of up to 12 months following sputum culture conversion, and recurrence or reinfection occurs in approximately 50% of cases (4, 25, 26). In a recent retrospective study of 297 patients with NTM, 40% of patients who had susceptibility results available were resistant to amikacin, ethambutol, or rifampin (4). The standard-of-care medications often are poorly tolerated and potentially toxic, especially when used in combination (13, 25). Thus, new systemic treatment options are needed, in particular for patients with refractory or drug-resistant NTM lung disease (25).

A potential limitation of this study was the limited exposure in healthy subjects to SPR720, in particular with respect to safety and tolerability. This study also did not evaluate SPR720 in combination with other drugs used to treat NTM. However, this was a first-in-human study with SPR720 that used a study design to satisfy regulatory requirements for determining doses and regimens to advance for future studies. In addition, the study evaluated the PK and tolerability in fasted versus fed states, as well as in an elderly cohort.

SPR720 is a chemically stable phosphate prodrug which converts rapidly to SPR719, the active moiety, *in vivo*. It belongs to a novel aminobenzimidazole class of antibacterial agents which target the ATPase subunits of gyrase and, when present, topoisomerase, resulting in growth inhibition of a range of pathogens (18). SPR719 is structurally and mechanistically distinct from, and demonstrates no cross-resistance with, fluoroquinolones. SPR719 is active *in vitro* against clinically relevant NTM pathogens, including isolates which are resistant to currently used agents (27). SPR720 represents a promising approach to the treatment of NTM-PD because of its novel mechanism of action and once-daily oral dosing. The tolerability profile of SPR720 in this first-in-human study was mostly confined to gastrointestinal complaints and headache. Results from this study provide evidence that SPR720 is generally well tolerated with once-daily dosing and the SPR719 PK characteristics are suitable for once-daily administration. This study supports further clinical evaluation of SPR720 in patients with NTM-PD.

## MATERIALS AND METHODS

This was a single-center, phase 1, randomized, double-blind, placebo-controlled, first-in-human study to assess the safety, tolerability, and PK of SPR720 (prodrug) and SPR719 (active moiety) following single or multiple ascending oral dose administrations of SPR720. This study was conducted according to the principles of the Declaration of Helsinki and Guidance on Good Clinical Practice. The study protocol, amendments, and informed consent forms were reviewed and approved by an Institutional Review Board. All subjects provided written informed consent prior to participating in any study activities. This study was registered at Clinicaltrials.gov under accession number NCT03796910.

### Study design.

This was a two-part multicohort study enrolling 96 healthy volunteers (including a healthy elderly cohort of subjects aged ≥65 years). Each cohort enrolled 8 subjects, randomized 3:1 to receive SPR720 (6 subjects) or placebo (2 subjects). Each subject was assigned to only one cohort.

Dose escalation between cohorts took place following blinded review by the study Safety Monitoring Group of all available safety, tolerability, and PK data from the preceding dose level.

**FIG 3:**
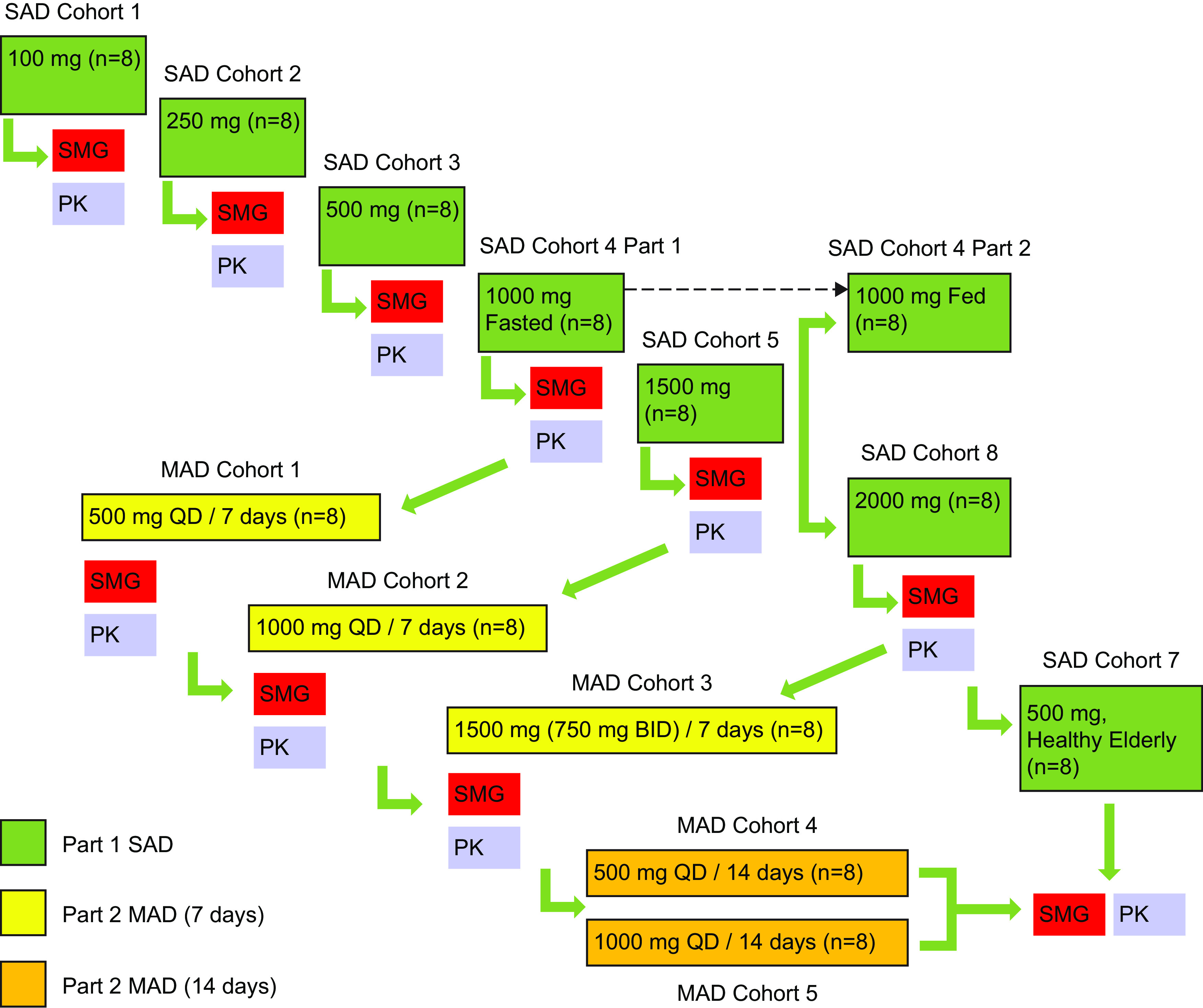
Study design. Subjects in SAD cohort 4, part 2, were dosed in the fed state; all others were fasting. BID, twice daily; MAD, multiple ascending dose; PK, pharmacokinetic; SAD, single ascending dose; SMG, safety monitoring group; QD, once daily.

SPR720 was supplied as 100- and 250-mg capsules for oral administration. Part 1 followed a single ascending dose (SAD) design in which six dose levels of SPR720 were evaluated (100, 250, 500, 1,000, 1,500, and 2,000 mg) in seven sequential cohorts, including a second 500-mg dose group of healthy elderly subjects aged ≥65 years (Fig. 3). Each cohort followed a schedule where two subjects (one randomized to placebo and one randomized to SPR720) were dosed in a blinded manner at least 24 h prior to the remainder of the cohort. In the absence of any safety concerns in these first two subjects, dosing of the remainder of the cohort proceeded on schedule. Subjects in SAD cohorts 1 to 5 received a single oral SPR720 or placebo after an overnight fast of at least 10 h. Fasting continued until 4 h postdose. Subjects in cohort 4 (food effect cohort; 1,000 mg) received an additional dose administered under fed conditions in which subjects were provided a standard U.S. Food and Drug Administration high-fat and high-calorie breakfast (28), with study drug administration occurring approximately 30 min after the start of the meal. Following review of interim data from cohort 4 fasted/fed periods, which suggested no clinically meaningful effect of food on PK and tolerability, dosing in subsequent SAD (cohorts 6 and 7) and MAD (cohorts 3 to 5) cohorts allowed for administration of SPR720 or placebo without respect to meals (nonfasting state), typically following a standardized meal or snack, for improved tolerability.

During part 2 (MAD), subjects received repeat dose administration of SPR720 (or placebo) for 7 days (cohorts 1 to 3) or 14 days (cohorts 4 and 5). All doses were administered once daily, with the exception of the 1,500-mg dose (MAD Cohort 3) that was administered as 750 mg q12h for improved tolerability based on review of data from SAD cohorts 5 (1,500 mg) and 6 (2,000 mg). Subjects in MAD cohorts 1 and 2 received SPR720 or placebo in the fasted state. Dosing in MAD cohorts 3 to 5 allowed for administration of SPR720 or placebo in a nonfasting state.

### Subject selection.

Adult male and female subjects aged 18 to 55 years (age ≥65 years for SAD cohort 7) who were assessed as medically healthy with no clinically significant abnormalities based on physical examination, vital signs (temperature, heart rate, blood pressure, and respiratory rate), ECG, and clinical laboratory testing (serum chemistry, hematology, and urinalysis) were eligible. Females were of nonchildbearing potential, and males used an acceptable form of contraception. Subjects were excluded for any clinically significant medical condition or laboratory abnormality; the presence or history of any clinically significant cardiac abnormalities, including clinically significant ECG abnormalities; a history of Clostridium difficile infection; positive human immunodeficiency virus (HIV) antibody, hepatitis B surface antigen (HBsAg) or hepatitis C antibody; a positive urine drug/alcohol test or history of substance or alcohol abuse, documented hypersensitivity, or anaphylaxis to any medication or use of tobacco or nicotine-containing products within 30 days; consumption of grapefruit-containing products with 7 days; receipt of any investigational drug or participation in a clinical trial within 30 days; or use of any prescription or over-the-counter medications with 7 days of randomization.

### Study assessments.

All AEs were coded using the Medical Dictionary for Regulatory Activities (MedDRA).

TEAEs were defined as existing conditions that worsened or events that occurred during the study after administration of study drug. Safety variables included clinical laboratory testing (hematology, serum chemistry, and urinalysis), vital signs (blood pressure, heart rate, body temperature, and respiratory rate), physical examination, and assessment of ECG parameters. Continuous Holter monitoring was performed in fasted cohorts (with baseline and postdose ECG extractions). Adverse events were recorded at each study visit.

### Pharmacokinetic analysis.

Pharmacokinetic parameters were evaluated using noncompartmental methods and included the maximum plasma concentration (*C*_max_), area under the concentration (AUC)-time curve from time zero to last measurable time point (AUC_0–_*_t_*) or from time zero to infinity (AUC_0–inf_), time to maximum concentration (*T*_max_), and terminal half-life (*t*_1/2_). In addition, the AUC-time curve from time zero to 12 or 24 h after the start of first dose (AUC_0–12_ and AUC_0–24_) was determined for MAD cohorts using noncompartmental methods. Data for AUC_0–last_ and AUC_0–inf_ were log transformed and analyzed using the trapezoidal rule.

In part 1 (SAD), blood samples for the determination of SPR720 and SPR719 plasma levels were obtained predose and 0.25, 0.5, 0.75, 1, 1.5, 2, 4, 8, 12, 24, 36, and 48 h postdose. Urine samples for determination of SPR719 urinary excretion were collected predose, and pooled urine collections took place over the following intervals: 0 to 4 h, 4 to 8 h, 8 to 12 h, and 12 to 24 h postdose.

In part 2 (MAD), blood samples for the determination of SPR720 and SPR719 plasma concentrations were obtained predose and at 0.25, 0.5, 0.75, 1, 1.5, 2, 4, 8, and 12 h postdose (days 1, 7, and 14, where applicable) and additionally at 24, 36, and 48 h after the last/final dose (day 7 or 14). Trough (24 h) samples were collected within 15 min prior to each dose on days 2 to 6 (7-day cohorts) and days 2 to 14 (14-day cohorts). Urine samples for determination of SPR719 urinary concentrations were collected predose on day 1 and pooled urine collections took place over the following intervals: 0 to 4 h, 4 to 8 h, 8 to 12 h, and 12 to 24 h postdose on day 1 (SAD and MAD cohorts) and 24 to 48 h after the final dose on day 7 and day 14 (MAD cohorts). Validated liquid chromatography-tandem mass spectrometry bioanalytical assays were used to determine SPR720 and SPR719 plasma concentrations and SPR719 urine concentrations. The lower limit of quantitation was 10 ng/ml for all the analytes in both matrices.

### Statistical analysis.

The safety population included all subjects who received study drug. The PK population included all subjects with evaluable concentration-time profiles for each active dose level who had no major protocol violations that impacted PK. Plasma concentrations and PK parameters for SPR719 were summarized for each treatment with Phoenix WinNonlin version 6.4. For PK calculations, concentration values below the limit of quantification were assigned a value of zero, and the actual time of sample collection was used.

**(i) Dose proportionality/independence.** For the SAD part of this study, dose proportionality was assessed by a regression analysis of the log-transformed *C*_max_, AUC_0–_*_t_*, and AUC_inf_ values versus the log-transformed dose using the power model with a fixed effect for log(dose). Dose independence was assessed for half-life by performing a regression analysis of the untransformed parameters versus dose with a fixed effect for dose. For each PK parameter, a point estimate and corresponding 95% confidence interval (CI) was calculated for the slope of the regression line. For the MAD part of the study, the dose proportionality assessment was performed separately for the fasted cohorts (cohorts 1 and 2) and fed cohorts (cohorts 3 to 5) using the same analysis method as described above. For day 1 and day 7, dose proportionality was assessed by performing a regression analysis of log-transformed *C*_max_ and AUC_0–12_ (day 7 only). The analysis was repeated for the additional MAD cohorts 4 and 5 at day 14 for *C*_max_ and AUC_0–12_.

**(ii) Food effect.** After logarithmic transformation, *C*_max_, AUC_0–_*_t_*, and AUC_inf_ values were analyzed with an analysis of variance (ANOVA), including fixed effects for treatment (fed or fasted) and subject. Point estimates and 90% CI values were constructed for the contrasts between treatments using the residual mean square error obtained from the ANOVA. The point and interval estimates were back-transformed to give estimates of the ratios of the geometric least-square mean (LS mean) and the corresponding 90% CI. Estimated geometric means are presented for each treatment.

**(iii) Steady state.** For each dose level, log-transformed trough concentration levels prior to the first dose each day (day 2 through to day 7/14) were analyzed with a mixed-effect ANOVA with the study day as a fixed effect and the subject as a random effect in order to establish whether and when steady-state had been attained for each dose level. Back-transformed ratios for the comparisons of each consecutive day (i.e., day 3/day 2) are presented along with the corresponding 90% CI.

**(iv) Accumulation.** For each MAD phase dose group, log-transformed *C*_max_, AUC_0–_*_t_*, and AUC_inf_ values on day 1 and day 7 (or day 14, where applicable) were analyzed with an ANOVA with the study day as a fixed effect and the subject as a random effect. For comparison, point estimates and 90% CI values for the difference between day 7/14 and day 1 were constructed using the residual mean-square error obtained from the ANOVA for each dose level. The point and interval estimates were back-transformed to give estimates of the ratios of the geometric least-square means and corresponding 90% CI values.
